# Persistence of antibody response 1.5 years after vaccination using 7-valent pneumococcal conjugate vaccine in patients with arthritis treated with different antirheumatic drugs

**DOI:** 10.1186/ar4127

**Published:** 2013-01-04

**Authors:** Meliha Crnkic Kapetanovic, Tore Saxne, Lennart Truedsson, Pierre Geborek

**Affiliations:** 1 Department of Clinical Sciences, Lund, Section of Rheumatology, Skåne University Hospital, Kioskgatan 3, Lund, SE-221 85 Sweden; 2 Department of Laboratory Medicine, Section of Microbiology and Immunology, Lund University, Entrégatan 5, Lund, SE-22 185 Sweden

## Abstract

**Introduction:**

The aim of this study was to explore the persistence of an antibody response 1.5 years after vaccination with 7-valent pneumococcal conjugate vaccine in patients with rheumatoid arthritis (RA) or spondyloarthropathy (SpA) treated with different antirheumatic drugs.

**Methods:**

Of 505 patients initially recruited, data on current antirheumatic treatment and blood samples were obtained from 398 (79%) subjects after mean (SD, range) 1.4 (0.5; 1 to 2) years. Antibody levels against pneumococcal serotypes 23F and 6B were analyzed by using enzyme-linked immunosorbent assay (ELISA). Original treatment groups were as follows: (a) RA receiving methotrexate (MTX); (b) RA taking anti-TNF monotherapy; (c) RA taking anti-TNF+MTX; (d) SpA with anti-TNF monotherapy; (e) SpA taking anti-TNF+MTX; and (f) SpA taking NSAID/analgesics. Geometric mean levels (GMLs; 95% CI) and proportion (percentage) of patients with putative protective antibody levels ≥1 mg/L for both serotypes, calculated in different treatment groups, were compared with results 4 to 6 weeks after vaccination. Patients remaining on initial treatment were included in the analysis. Possible predictors of persistence of protective antibody response were analysed by using logistic regression analysis.

**Results:**

Of 398 patients participating in the 1.5-year follow up, 302 patients (RA, 163, and SpA, 139) had unchanged medication. Compared with postvaccination levels at 1.5 years, GMLs for each serotype were significantly lower in all groups (*P *between 0.035 and <0.001; paired-sample *t *test), as were the proportions of patients with protective antibody levels for both serotypes (*P *< 0.001; χ^2 ^test). Higher prevaccination antibody levels for both serotypes 23F and 6B were associated with better persistence of protective antibodies (*P *< 0.001). Compared with patients with protective antibody levels at 1.5 years, those not having protective antibody levels were older, more often women, had longer disease duration and higher HAQ and DAS, and had a lower proportion of initial responders to both serotypes.

Concomitant anti-TNF treatment and MTX were identified as negative predictors of the persistence of protective antibodies among RA patients (*P *= 0.024 and *P *= 0.065, respectively). Only age 65 years or older (*P *= 0.017) and not antirheumatic treatment was found to be a negative predictor of protective antibodies in patients with SpA.

**Conclusions:**

After initial increase, 1.5 years after pneumococcal vaccination with 7-valent conjugate vaccine, postvaccination antibody levels decreased significantly, reaching levels before vaccination in this cohort of patients with established arthritis treated with different antirheumatic drugs. MTX and anti-TNF treatment predicted low persistence of protective immunity among patients with RA. To boost antibody response, early revaccination with conjugate vaccine might be needed in patients receiving potent immunosuppressive remedies.

**Trial registration number:**

EudraCT EU 2007-006539-29 and NCT00828997.

## Introduction

Infections are a well-recognized cause of increased morbidity and mortality in patients with inflammatory rheumatic diseases, partly because of the use of potent immunomodulating drugs [1-5]. According to recently published European League Against Rheumatism evidence-based recommendations for vaccination, pneumococcal vaccination should be strongly considered for all adult patients with rheumatic diseases [6]. Currently available 23-valent pneumococcal polysaccharide vaccine covers 85% to 90% of the serotypes causing invasive pneumococcal disease, such as pneumonia, pneumococcal bacteremia, or meningitis [7]. However, a suboptimal rate of pneumococcal vaccination among rheumatology outpatients receiving different immunosuppressive remedies has been reported [8].

Several reasons are found for low pneumococcal vaccination coverage in these patients. One important issue is the limited evidence of efficacy of the polysaccharide vaccine in preventing infections in this high-risk group of patients, although a recent study reported an increased risk of developing pneumonia in pneumococcal nonvaccinated compared with vaccinated rheumatoid arthritis (RA) patients receiving methotrexate (MTX) (180 patients followed up for 10 years) [9]. Larger controlled studies confirming these findings are lacking.

The majority of studies of patients with rheumatic diseases, including those in our earlier reports, investigated short-term antibody responses after pneumococcal vaccination as surrogate markers of vaccination efficacy [10,11]. Persistence of protective antibodies over time is considered an indicator of remaining protection against infection [12-14]. A Finnish study showed initial good antibody response but returned to close to prevaccination levels within 3 years after pneumococcal vaccination in an elderly population [15]. In contrast, Mucher *et al*. [16] reported that both primary vaccination and revaccination induced antibody responses persisting longer than 5 years in middle-aged and elderly people [16]. Corresponding studies with immunocompromised patients also report conflicting results. Systemic lupus erythematosus patients had a low persistence of protective antibodies at years 1, 2, and 3 after pneumococcal vaccination compared with healthy controls [17]. Similarly, antibody levels declined 3 years after vaccination in patients with chronic pulmonary diseases, HIV-infected individuals, and renal-transplant recipients [18-20]. Conversely, Coulson *et al*. [9] found elevated antibody levels to most pneumococcal polysaccharides for up to 10 years after vaccination in RA patients receiving MTX. Reports on the impact of newer antirheumatic treatments such as tumor necrosis factor (TNF) blockers on the persistence of protective antibodies are lacking. Likewise, the possible advantages of pneumococcal conjugate vaccine on the duration of protective immunity in adult patients with established arthritis have not been investigated.

The aim of the present study was to investigate the 1.5-year persistence of serotype-specific antibodies after conjugate pneumococcal vaccination in patients with RA and spondyloarthropathy (SpA) treated with different antiinflammatory remedies, including TNF blockers, compared with spondyloarthropathy patients treated with NSAID/analgesics. We also wanted to study the influence of demographics, disease, and treatment characteristics on protective antibody levels.

## Materials and methods

In total, 505 patients with established arthritis (RA, 253, and SpA, 252) were initially vaccinated with a single dose of 0.5 ml of 7-valent pneumococcal conjugate vaccine intramuscularly, as previously reported [11]. Based on diagnosis and treatments, patients were divided into six predefined groups. The groups were as follows: 1. RA on MTX (n = 85); 2. RA on anti-TNF as monotherapy (n = 79); 3. RA on anti-TNF+MTX (n = 89); 4. SpA on anti-TNF monotherapy (n = 83); 5. SpA on anti-TNF+MTX (n = 83), and 6. SpA on NSAID/analgesics (n = 86). Only patients taking unchanged antirheumatic treatments for at least 4 weeks before vaccination and 4 to 6 weeks after vaccination were eligible for the study. Patients were offered inclusion in a follow-up study that included collecting data on current medication and blood-sample drawing. Of the original 505 vaccinated patients, 398 participated in the 1.5-year follow-up. The mean (SD; range) time between vaccination and follow-up visit was 1.4 (0.5; 1 to 2) years. Patients who, at the 1.5-year follow-up, remained on the initial treatment were eligible for the present study. Antibody levels against two pneumococcal polysaccharide antigens (23F and 6B) were measured by using standardized ELISA, as previously described [21]. All analyses were performed at the same immunology department and by using the same method.

Geometric mean antibody levels (GMLs) for each serotype were calculated and compared with those at vaccination and 4 to 6 weeks after vaccination. Antibody levels ≥1 mg/L are considered putative protective, and this level was chosen as protective in the current study [12,13].

Ethical approval for the vaccination (file number 97/2007) and additional ethical approval for the follow-up study (file number 5019/2009) were obtained from the Ethical Review Board at Lund University. Written informed consent was obtained from all participants before inclusion in the study. The study was conducted as an investigator driven clinical trial, registered on line with numbers EudraCT 2007-006539-29 and NCT00828997, and approved by the Swedish medical products agency (MPA; Läkemedelsverket; file number 151: 2007/88047).

### Statistics

GMLs (95% CI) were calculated from natural logarithm transformed values of antibody levels. Comparisons between GMLs were performed using paired samples *t *test. A χ^2 ^test was used to compare the proportion of patients with protective antibody levels for each serotype. In general, the χ^2 ^test was used for comparison between categoric variables and the Mann-Whitney *U *test for continuous ones. Possible predictors of protective antibody levels for all patients included in the study, and RA and SpA separately, were analyzed by using three different univariate and multivariate logistic regression models.

## Results

Of 398 patients participating in the 1.5-year follow-up, 302 patients (RA, 163; and SpA, 139) did not change their antirheumatic treatment during follow-up, and these patients were included in the analysis.

Number (percentage) and characteristics of patients remaining on the original antirheumatic treatment at 1.5 years of follow-up are shown in Table 1.

**Table 1:** Demographic and disease characteristics of patients receiving the same treatment at vaccination and at 1

	RA (*n *= 163)			SpA (*n *= 139)		
Treatment group	**MTX monotherapy**	**Anti-TNF monotherapy**	**Anti-TNF + MTX**	**Anti-TNF monotherapy**	**Anti-TNF + MTX**	**NSAID/analgesics**

Number of patients at vaccination	85	79	89	83	83	86

Number (%) of patients remaining on original therapy at 1.5-year follow-up	**57 (67%)**	**50 (63%)**	**56 (63%)**	**47 (47%)**	**49 (59%)**	**43 (50%)**

Age, mean (SD); years	63.5 (11)	59.9 (14)	60.5 (9)	50.3 (12)	51.6 (11)	52.8 (12)

Female (%)	77%	90%	75%	38%	57%	49%

Disease duration, mean (SD); years	12.6 (10)	19.8 (11)	16.8 (11)	16.8 (11)	13.1 (10)	13.6 (12)

RF positive (%)	81	86	84	-	-	-

Anti-CCP positive (%)	77	76	88	-	-	-

HLA B27 positive (%)	-	-	-	53	33	63

**Figure 1:**
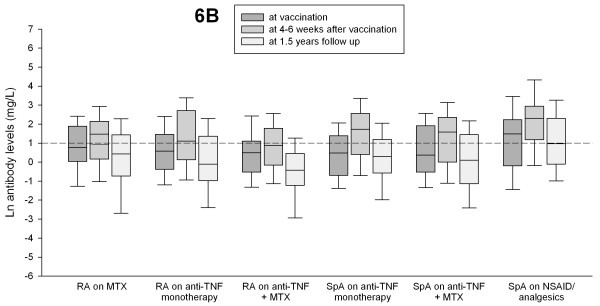
**Antibody levels for serotype 6B at vaccination, at 4 to 6 weeks after vaccination, and at 1.5-year follow-up in patients with rheumatoid arthritis (RA) and spondyloarthropathy (SpA) treated with different antiinflammatory drugs**. Antibody levels ≥1 mg/L were considered putative protective (short dashed line).

**Figure 2:**
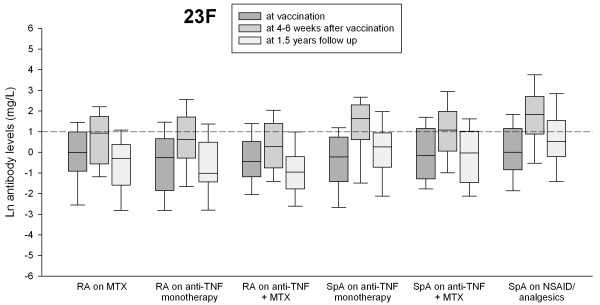
**Antibody levels for serotype 23F at vaccination, at 4to 6 weeks after vaccination, and at 1.5-year follow-up in patients with rheumatoid arthritis (RA) and spondyloarthropathy (SpA) treated with different antiinflammatory drugs**. Antibody levels ≥1 mg/L were considered putative protective (short dashed line).

Table 2 summarizes GML (95% CI) in milligrams per liter and percentage of patients with protective antibody levels for 23F and 6B in different treatment groups at vaccination, at 4 to 6 weeks, and at 1.5-year follow-up. In all treatment groups, GMLs for each serotype were significantly lower at 1.5-year follow-up compared with the GML 4 to 6 weeks after vaccination (*P *between 0.035 and <0.001; paired-sample *t *test; Figures 1 and 2), as were the proportions of patients with protective antibody levels for both serotypes (*P *< 0.001; χ^2 ^test).

**Table 2:** Geometric mean level (GML; 95% CI) in milligrams per liter and percentage of patients with protective antibody levels for both 23F and 6B in different treatment groups at vaccination, at 4 to 6 weeks, and at 1

	RA on methotrexate	RA on anti-TNF as monotherapy	RA on anti-TNF+MTX	SpA on anti-TNF as monotherapy	SpA on anti-TNF+MTX	SpA on NSAID/analgesics
At vaccination						

Patient number (*n*)	85	79	89	83	83	**86**

GML (95% CI) for 23F	0.7 (0.5-1.1)	0.6 (0.4-0.8)	0.7 (0.5-0.9)	0.7 (0.5-0.9)	0.8 (0.6-1.2)	0.97 (0.7-1.4)

GML (95% CI) for 6B	2.0 (1.4-2.8)	1.4 (0.9-2.0)	1.5 (1.1-2.9)	1.5 (1.1-2.1)	1.7 (1.1-2.5)	2.9 (2.1-4.0)

4 to 6 weeks of follow-up						

GML (95% CI) for 23F	1.9 (1.3-2.6)	1.9 (1.3-2.7)	1.4 (1.1-1.9)	3.1 (2.2-4.5)	2.5 (1.8-3.5)	6.4 (4.5-9.1)

GML (95% CI) for 6B	3.5 (2.5-4.9)	3.6 (2.5-5.3)	2.3 (1.7-3.2)	4.8 (3.3-6.9)	3.0 (22.1-4.4)	9.5 (6.7-13.6)

Percentage of patients with protective antibody levels for both 23F and 6B	67%	58%	52%	78%	65%	84%

1.5-year follow-up						

Patient number (*n*)	57	50	56	47	**49 **	**43 **

GML (95% CI) for 23F	0.6 (0.4-0.8)	0.5 (0.3-0.8)	0.4 (0.3-0.5)	1.0 (0.6-1.6)	0.8 (0.5-1.3)	2.0 (1.3-3.0)

GML (95% CI) for 6B	1.2 (0.7-1.9)	1.0 (0.6-1.7)	0.6 (0.4-1.0)	1.4 (0.9-2.1)	1.0 (0.6-1.6)	2.8 (1.7-4.6)

Percentage of patients with protective antibody levels for both 23F and 6B	40%	32%	20%	60%	49%	70%

1.5-year follow-up/4 to 6 weeks of follow-up						

Relative ratio of protective antibody levels	0.61	0.55	0.38	0.77	0.75	0.84

GML at 1.5-year follow-up was also lower compared with prevaccination antibody levels for each serotype in all treatment groups, but differences were significant only for RA patients taking anti-TNF+MTX.

**Figure 3:**
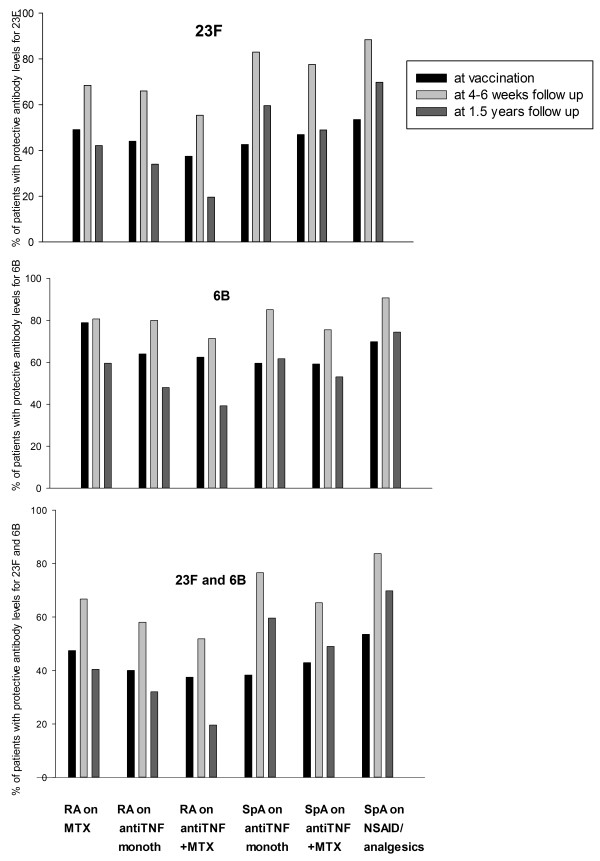
**Percentage of patients with putative protective antibody levels (≥1 mg/L) for serotype 23 F 6B and both 23 F and 6 B (C) at vaccination, at 4 to 6 weeks after vaccination, and at 1.5-year follow-up in patients with rheumatoid arthritis (RA) and spondyloarthropathy (SpA) treated with different antiinflammatory drugs**.

SpA patients taking NSAIDs/analgesics (that is, not treated with immunosuppressive remedies) had significantly higher proportions of patients with protective antibody levels both at 4 to 6 weeks and at 1.5 years of follow-up. Although the proportion of patients with protective antibody levels decreased in this group at 1.5-year follow-up, the relative ratio of protective antibody levels (that is, 1.5-year follow-up/4 to 6 weeks follow-up) was still highest in SpA patients with NSAIDs/analgesics. The lowest relative ratio of protective antibody levels was seen in RA patients taking anti-TNF+MTX and in general lower in RA patients than in patients with SpA. The proportions of patients with protective antibody levels for 23F, 6B, and both serotypes at vaccination, at 4 to 6 weeks after vaccination, and at 1.5-year follow-up in different treatment groups are shown in Figure 3.

Clinical and demographic characteristics of patients after splitting according to protective antibody levels 1.5 years after vaccination (yes/no) are shown in Table 3. Patients without protective antibodies were on average older, more frequently women with RA of longer disease duration, and higher HAQ and DAS at vaccination. These patients were more often treated with MTX, anti-TNF, and had concomitant prednisolone treatment. Protective antibody levels for both serotypes at vaccination, as well as a positive antibody response for both serotypes tested, were associated with protective antibody levels at 1.5 years (Table 3).

**Table 3:** Disease and demographic characteristics of patients based on the status of protective antibody levels at 1

	Patients with protective antibody levels at 1.5 years of follow-up (*n *= 132)	Patients without protective antibody levels at 1.5 years of follow-up (*n *= 170)	*P *value
Age (years); mean (SD) (range)	51.7 (13) (24-79)	60.1 (11) (30-85)	***P *< 0.001^a^**

Patient age ≥65 years (%)	16%	37%	***P *< 0.001**^b^

Gender (female %)	58%	72%	***P *= 0.01**^b^

Disease duration (years); mean (SD) (range)	13.8 (10) (0-45)	16.7 (11) (0-46)	***P *= 0.039^a^**

DAS at vaccination; mean (SD) (range)	3.0 (1.3) (0-5.6)	3.5 (1) (0-6.4)	***P *= 0.001^a^**

HAQ at vaccination; mean (SD) (range)	0.6 (0.5) (0-2)	0.9 (0.7) (0-3)	***P *< 0.001^a^**

RA (yes, %)	38/62	66/34	***P *< 0.001**^b^

Ongoing MTX (yes, %)	44%	61%	***P *< 0.001**^b^

Ongoing anti-TNF treatment (yes, %)	60%	72%	***P *= 0.029**^b^

Positive antibody response for 6B and 23F at 4 to 6 weeks of follow-up (yes, %)	37.1%	25%	***P *= 0.027**^b^

Protective antibody levels for both 6B and 23F serotypes at vaccination (%)	72%	20.6%	***P *< 0.001**^b^

**^a^**Mann-Whitney *U *test. ^b^χ^2 ^test.

### Predictor analysis

Results of univariate and multivariate regression analysis for patients with RA and SpA separately are summarized in Table 4. After adjustments for demographic and disease characteristics at vaccination, concomitant anti-TNF treatment and treatment with MTX were identified as negative predictors of persistence of protective antibody levels for both serotypes tested (*P *= 0.024 and 0.065, respectively). Age 65 years or older but not immunosuppressive treatment had a significant negative impact on the persistence of protective immunity in SpA patients (*P *= 0.012). Higher prevaccination antibody levels for both serotypes was a strong significant predictor of the persistence of protective antibodies both among RA and SpA patients (*P *< 0.001).

**Table 4:** Predictors of persistence of protective antibody levels for both 23F and 6B 1.5 years after pneumococcal vaccination by using 7-valent conjugate vaccine in patients with rheumatoid arthritis and spondyloarthropathy

Univariate logistic regression analysis		
	**RA** ***P *value; OR (95% CI)**	**Spondyloarthropathy** ***P *value; OR, 95% CI**

Age ≥65 years (yes/no)	*P *= 0.144; OR 0.59 (0.29-1.2)	*P *= 0.012; OR 0.24 (0.08-0.74)

Gender (male/female)	*P *= 0.727; OR 0.86 (0.37-2.0)	*P *= 0.120; OR 1.72 (0.07-3.40)

Disease duration (years)	*P *= 0.011; OR 0.96 (0.93-0.99)	*P *= 0.895; OR 1.0 (0.97-1.03)

DAS at vaccination (0-6.5)	*P *= 0.684; OR 0.94 (0.68-1.28)	*P *= 0.014; OR 0.70 (0.51-0.93)

HAQ at vaccination (0-3)	*P *= 0.020; OR 0.54 (0.32-0.91)	*P *= 0.043; OR 0.48 (0.24-0.98)

Ongoing MTX (yes/no)	*P *= 0.807; OR 0.92 (0.45-1.9)	*P *= 0.078; OR 0.53 (0.26-1.07)

Ongoing anti-TNF (yes/no)	*P *= 0.066; OR 0.53 (0.27-1.04)	*P *= 0.086; OR 0.51 (0.24-1.1)

Ongoing prednisolone (yes/no)	*P *= 0.259; OR 0.64 (0.29-1.40)	*P *= 0.390; OR 0.62 (0.21-1.84)

Positive antibody response for both 6B and 23F at 4 to 6 weeks	*P *= 0.180; OR 1.70 (0.78-3.71)	*P *= 0.630; OR 1.19 (0.59-2.36)

Protective prevaccination antibody levels for both 23F and 6B (yes/no)	*P *< 0.001; OR 12.1 (5.38-27.4)	*P *< 001; OR 14.6 (5.83-36.4)

Multivariate logistic regression analysis		

Age ≥65 years (yes/no)	*P ***= **0.188; OR 0.59 (0.26-1.30)	***P *= 0.017; OR 0.22 (0.06-0.76)**

Gender (male/female)	*P ***= **0.480; OR 0.70 (0.26-1.88)	*P ***= **0.637; OR 1.24 (0.51-2.97)

Disease duration (years)	*P ***= **0.214; OR 0.98 (0.94-1.01)	*P ***= **0.873; OR 1.01 (0.97-1.04)

HAQ at vaccination (0-3)	*P ***= **0.139; OR 0.62 (0.32-1.17)	*P ***= **0.291; OR 0.63 (0.27-1.49)

Ongoing MTX (yes/no)	***P *= 0.065; OR 0.39 (0.14-1.06)**	*P ***= **0.882; OR 0.94 (0.39-2.27)

Ongoing anti-TNF (yes/no)	***P *= 0.024; OR 0.34 (0.13-0.87)**	*P ***= **0.195; OR 0.54 (0.21-1.37)

Positive antibody response for both 6B and 23F at 4-6 weeks	*P ***= **0.388; OR 1.45 (0.62-3.40)	*P ***= **0.916; OR 1.04 (0.49-2.23)

Adjusted for age older than 65 years, gender, disease duration, HAQ, ongoing MTX, ongoing anti-TNF, and positive antibody response for both serotypes tested. MTX, methotrexate; HAQ, health-assessment questionnaire.

Analysis of patients who changed their antirheumatic treatment during follow-up

Altogether, 96 of 398 patients participating in the 1.5-year follow-up changed their antirheumatic treatment (RA, 51; and SpA, 45). Mean age and disease duration (SD) among these RA patients was 59.8 (13.5) and 15.7 (12.7) years, respectively, and 82.4% were women. Of these 32 patients, 62.7% had protective antibodies for both serotypes 4 to 6 weeks after vaccination, but only 13 (20.5%) patients had this at 1.5-year follow-up.

Mean age and disease duration (SD) among SpA patients who had changed their medication at the 1.5-year follow-up were 51 (12.7) and 13.4 (11.4) years, respectively, and 46.7% were women. Of these SpA patients, 34 (75.6%) patients had protective antibody levels for both serotypes 4 to 6 weeks after vaccination, whereas only 18 (40.0%) patients had this at 1.5-year follow-up.

RA and SpA patients who switched their antirheumatic treatment had lower proportions of protective antibodies at 1.5-year follow-up, but no significant differences was seen in other demographic and disease characteristics compared with RA and SpA patients remaining on the original treatment.

## Discussion

The main finding in the present report is the low persistence of protective immunity against the serotypes tested in arthritis patients compared with those reported in healthy individuals 1 to 2 years after vaccination [16]. Higher age was associated with lower protective antibody levels in both RA and spondyloarthropathy patients, regardless of antirheumatic treatment. In general, RA patients had lower persistence of protective antibodies compared with SpA patients. The effect of immunosuppressive treatments, including both MTX and anti-TNF drugs on 1.5-year antibody levels, was less clear as compared with the results 4 to 6 weeks after vaccination, whereas MTX clearly hampered this response [11].

Our findings of a rapid decline to, and in RA patients taking anti-TNF and MTX, prevaccination levels already 1.5 years after a single dose of conjugate pneumococcal vaccine is problematic. However, serum antibody levels are surrogate markers of vaccine protection, but measuring truly bactericidal antibodies is challenging [12-14]. The correlation between serum antibody levels and protection against infections caused by *Streptococcus pneumoniae *varies for different populations, serotypes, and infections [12]. A decline of postvaccination antibody levels probably results in decreased protection against infections [14]. A rapid decrease in the antibody levels after pneumococcal vaccination with 23-valent polysaccharide vaccine has been reported in subgroups of elderly or immunocompromised individuals [14,15,18-20]. Corroborating results of conjugate pneumococcal vaccination in immunosuppressed patients with inflammatory rheumatic diseases are lacking, and the effect of repeated vaccination procedures also needs further studies.

We also found that patients 65 years or older had lower levels of protective antibodies for both serotypes compared with younger individuals, regardless of treatment. This could be an effect of age-related decline in immune responses (immunosenescence), which also includes impaired ability for B cells to respond to new antigen challenges [22]. We were unable to detect a statistical interaction between drug treatment and age, suggesting no specific age-related drug interaction (that is, an increased sensitivity to drug-induced immunosuppression) did not seem to occur in old individuals, other than immunosenescence. However, a possible impact of RA disease itself on vaccine response cannot be ruled out.

Both MTX and anti-TNF treatment were negative predictors of the persistence of protective immunity at 1.5 years when analyzed among all patients (Table 4). When we analyzed RA patients separately, concomitant anti-TNF treatment remained a negative predictor, whereas MTX showed only a trend in RA. In SpA patients 65 years or older, a negative effect on persistence of protective antibodies appeared, but treatment did not affect antibody persistence significantly. Overall, more SpA patients had protective antibodies levels for each serotype compared with the corresponding RA treatment groups. Expectedly, SpA patients taking NSAIDs/analgesics without concomitant immunosuppressive drugs had the highest persistence of protective antibodies, although not statistically different from SpA on anti-TNF monotherapy. Patients with SpA participating in the present study were younger; a larger proportion were men; they had longer disease duration, and one third were not treated with immunosuppressive remedies, which all may have contributed to these diverging results. Because RA diagnosis itself was associated with a lower persistence of protective antibodies, the difference in immunologic disturbance as a part of each disease may play a role.

Furthermore, we previously reported that prednisolone improved antibody response 4 to 6 weeks after pneumococcal vaccination in this cohort [11]. However, neither use of prednisolone nor dosage of prednisolone had any significant impact on the persistence of protective antibodies after 1.5 years. These results are consistent with previously reported data on the influence of systemic steroids on immunogenicity of pneumococcal polysaccharide vaccine in patients with chronic obstructive lung disease 12 month after the vaccination [23].

Results from the present study are in line with some previously published data, including those from other immunocompromised patients, such as HIV-infected patients or renal transplant recipients, in whom antibody response after pneumococcal vaccination with either polysaccharide or conjugate vaccine was shown to decline significantly by 3 years [19,20]. In contrast, sustained antibody levels up to 10 years after pneumococcal vaccination in 124 consecutive RA patients taking MTX have recently been reported [9]. Patients participating in that study received the vaccine early in the course of RA and were significantly younger at vaccination. All participants were taking MTX at vaccination and had a stable MTX dose for at least a year before enrolment in the study, occurring approximately 10 years after vaccination. Furthermore, it seems that none of the patients in that study had received concomitant DMARDs or biologics. This indicates that the study population represents a selection of younger patients with early RA sufficiently treated with MTX and with good compliance with the treatment, making the results less comparable with those in our report.

Regardless of treatment and diagnosis, we found that high prevaccination antibody levels predicted a better persistence of protective immunity at the 1.5-year follow-up. This is consistent with a previously reported association between high prevaccination antibody levels and better vaccination response [24]. The high prevaccination antibody levels are most likely due to natural pneumococci exposure or cross-reacting bacteria in vaccine-naive patients.

Patients participating in this study received a dose of 7-valent conjugate polysaccharide vaccine. Through conjugation of pneumococcal polysaccharide antigen to a polypeptide carrier protein, this vaccine stimulates B-cell production of antibodies and their development into memory cells. However, we could not see that this pneumococcal conjugate vaccine has any advantage over 23-polysaccharide vaccine, either on antibody production of serotype-specific antibodies or on the persistence of protective antibodies in immunosuppressed patients with arthritis. Conversely, we did not test whether revaccination would have any effect on memory cells [25].

The group of patients that changed the antirheumatic treatment during the follow-up period was relatively small and could not be analyzed in detail. However, overall, the frequency of protective antibodies was quite low at 1.5-year follow-up in both RA and SpA patients, suggesting that this group did benefit to a lesser degree from the vaccination.

In the immunology laboratory where the analysis of antibody levels was performed, antibody levels ≥1 mg/L are considered protective. These antibody levels were shown to correlate with age-dependent efficacy of polysaccharide vaccines [13]. However, the relation between antibody levels and protection against pneumococcal disease is a controversial issue [12]. Because no general recommendations for protective antibody levels in adults are available, the clinical significance of the declines in detectable antibodies is not clear. This remains a limitation of the present study. Nevertheless, antibody levels decreasing to those before vaccination suggest lack of protection already after 1.5 years.

Revaccination against invasive pneumococcal diseases by using 23-valent pneumococcal polysaccharide vaccine is recommended as early as 5 years after primary vaccination [6,7]. Our data indicate that revaccination earlier than recommended may be needed in patients with established arthritis.

## Conclusions

The persistence of protective immunity was low at 1.5 years after vaccination with pneumococcal conjugate vaccine in patients with established arthritis treated with different antirheumatic drugs. To boost antibody response, early revaccination with conjugate vaccine might be needed in arthritis patients receiving potent immunosuppressive remedies.

## Abbreviations

GML: geometric mean level; MTX: methotrexate; NSAID: nonsteroidal antiinflammatory drug; RA: rheumatoid arthritis; SpA: spondyloarthropathy; TNF: tumor necrosis factor.

## Competing interests

Prevenar® vaccine for this study was provided by Pfizer, NewYork, USA. All authors declare no conflict of interest.

## Authors' contributions

MCK participated in the design of the study, performed the statistical analysis, and wrote the manuscript. TS, LT, and PG conceived of the study, participated in its design and coordination, and helped to draft the manuscript. All authors read and approved the final manuscript.
